# Carvacrol as a one health–relevant antimicrobial agent: mechanistic insights on its impact on gnotobiotic Artemia and Vibrio campbellii interactions

**DOI:** 10.3389/fimmu.2026.1797445

**Published:** 2026-04-10

**Authors:** Kartik Baruah, Dipesh Debnath, Prathibha Patabandige Sarath Kumara Patabandi, Peter Bossier, Qiang Yang, Tom Defoirdt, Parisa Norouzitallab

**Affiliations:** 1 Department of Applied Animal Science and Welfare, Faculty of Veterinary Medicine and Animal Sciences, Swedish University of Agricultural Sciences, Uppsala, Sweden; 2 Laboratory of Aquaculture & Artemia Reference Center, Department of Animal Sciences and Aquatic Ecology, Faculty of Bioscience Engineering, Ghent University, Gent, Belgium; 3 Central Inland Fisheries Research Institute, Regional Centre, Housefed Complex, Guwahati, India; 4 Department of Livestock Production, Faculty of Agricultural Sciences, Sabaragamuwa University of Sri Lanka, Belihuloya, Sri Lanka; 5 Center for Microbial Ecology and Technology (CMET), Department of Biotechnology, Ghent University, Gent, Belgium

**Keywords:** carvacrol, mutl-drug resistance, one-health, shrimp aquaculture, *Vibrio campbellii*

## Abstract

**Introduction:**

Bacteria belonging to the Harveyi clade of vibrios, including *Vibrio campbellii* infect many wild and cultured aquatic organisms and cause major losses in global aquaculture. Although not a primary human pathogen, *V. campbellii* can act opportunistically, particularly following exposure to marine environments or seafood, and in immunocompromised individuals, highlighting the links between aquatic ecosystems, food production systems, and human health. Carvacrol, a phenolic monoterpenoid found in oregano and thyme essential oils, is approved for use in human and animal food. Beyond its safety profile, this compound has also been reported to possess diverse pharmacological effects, including anticarcinogenic, anti-inflammatory and antimicrobial.

**Methods:**

Using gnotobiotic brine shrimp *Artemia* as an *in vivo* model, we examined whether carvacrol can provide dual protection against *V. campbellii* by inhibiting the production of bacterial virulence and modulating host immune responses.

**Results and discussion:**

Carvacrol significantly improved the survival of *Artemia* during *V. campbellii* challenge while showing low toxicity at effective concentrations. The protection was associated with reduced bacterial virulence, including decreased biofilm formation and lower hemolytic and caseinase activities. Additionally, carvacrol modulated the expression of defence-related genes (*hsp70*, *prophenoloxidase*, *transglutaminase*, and *ferritin*) in time-dependent and stochastic patterns, rather than sustained upregulation. Overall, these findings suggest that carvacrol enhances disease resistance through both pathogen- and host-directed mechanisms. Given its food-grade safety status, carvacrol holds strong translational potential as a functional antimicrobial strategy to support disease control and health management in aquaculture, warranting further evaluation under realistic farming conditions.

## 1 Introduction

Aquaculture is the fastest-growing form of food production around the globe and currently provides over 50% of fish for human consumption worldwide (1). To meet the increasing demand for animal protein and economic requirements, aquaculture practices have become increasingly intensive. However, this intensification has resulted in a marked increase in infectious diseases, particularly bacterial diseases, which have been estimated to cause economic losses exceeding USD 6 billion per year (2). Among the major bacterial pathogens affecting (marine) aquaculture systems is *Vibrio campbellii*, a curved rod-shaped Gram-negative bacterium belonging to the Harveyi clade of vibrios. This opportunistic pathogen infects a wide range of wild and cultured aquatic organisms, leading to huge losses in the aquaculture industry worldwide (3). Of particular concern, certain *V. campbellii* strains harbor a specific toxin-encoding plasmid associated with causing Acute hepatopancreatic necrosis disease (AHPND) (4). The shrimp farming industry in Asia alone faces annual losses exceeding 1 billion dollars due to this disease (2).

Beyond its impact on aquatic animal health, *V. campbellii* is increasingly recognized as a One Health–relevant bacterium, reflecting the interconnectedness of aquatic ecosystems, food production systems, and human health (5, 6). Although it is not considered a primary human pathogen, *V. campbellii* has been reported as an opportunistic agent in human infections, particularly following exposure to marine environments or seafood, and in immunocompromised individuals (7, 8). Moreover, it shares conserved virulence traits and close phylogenetic relatedness with clinically important zoonotic vibrios (5, 9, 10). Importantly, aquaculture environments can serve as reservoirs for antimicrobial-resistant *Vibrio* spp., facilitating the dissemination of resistance determinants through the aquatic environment and food chain, thereby posing potential risks to human health (6, 11).

To combat bacterial diseases, the conventional approach involves the use of antibiotics and chemical disinfectants. However, the extensive and often indiscriminate use of these chemotherapeutic agents has led to a rapid decline in their efficacy. More critically, these traditional antimicrobials have driven the selection, emergence and spread of multidrug-resistant pathogens, posing a significant threat to both the environment and the health of animals and humans (6). From a One Health perspective, the emergence of multidrug-resistant pathogens in aquaculture systems represents a global concern (6). The diseases caused by antibiotic-resistant bacteria are currently one of the leading causes of death in both farmed animals and humans worldwide, and this situation is predicted to worsen in the near future (12, 13). These challenges underscore the urgent need for sustainable, effective and environmentally compatible alternatives to conventional antimicrobials to control bacterial pathogens in the aquaculture industry.

There is currently a surge in interest in using natural antibacterial compounds derived from plant sources. A notable example is carvacrol, a phenolic monoterpenoid present in the essential oils of several herbs, such as oregano, thyme, pepperwort, and wild bergamot (14–17). Carvacrol has received safety approvals for use in food from the Council of Europe (2000), the FAO/WHO Committee on Food Additives (2001), and the U.S. Food and Drug Administration (2010). Beyond its safety profile, this compound has also been reported to possess diverse pharmacological effects, including anticarcinogenic, anti-inflammatory and antimicrobial properties (18). Studies have shown that carvacrol exhibits antimicrobial activities against numerous microbial strains, including both Gram-positive and Gram-negative bacteria, as well as fungi (18). For example, in Gram-positive bacteria *Listeria monocytogenes*, carvacrol was shown to significantly inhibit biofilm formation and virulence by reducing extracellular polymeric substance production, impairing bacterial motility, suppressing hemolytic activity, and down-regulating key genes associated with biofilm development and pathogenicity (19). In another study, spray-dried microencapsulation of carvacrol markedly enhanced its activity against Gram-negative bacteria *Pseudomonas aeruginosa*, reducing the minimum inhibitory concentration fourfold and eliminating *P. aeruginosa* biofilms below detectable levels within 15 minutes by disrupting the bacterial cell membrane (20). Carvacrol has been demonstrated to disrupt the integrity of cytoplasmic membranes, subsequently inhibiting ATP synthesis and thereby reducing energy-dependent cellular processes, such as the synthesis of enzymes and toxins (21–23). In addition to antibacterial activities, phenolic compounds have also been reported to inhibit the production of virulence factors (24). However, the impact of carvacrol on virulence factor production in *V. campbellii* has not yet been studied. In parallel, growing evidence suggests that carvacrol also modulates host immune responses, thereby enhancing host resilience to infection (25, 26). Indeed, our previous research demonstrated the potential of carvacrol as a prophylactic agent in the brine shrimp *Artemia* model. Our results provided strong evidence that carvacrol triggers the production of heat shock protein 72 in *Artemia*, thereby enhancing its ability to resist environmental stress or bacterial infection (27).

Building on these findings and adopting a One Health–oriented framework, the present study employs a gnotobiotic *Artemia* model to dissect the dual role of carvacrol as both an antimicrobial and a host-protective agent against *V. campbellii*. Specifically, we investigated the impact of carvacrol on the production of key virulence factors in *V. campbellii* and its modulatory effects on immune-related gene expression in *Artemia*. By providing mechanistic insights into host–pathogen interactions, this study aims to evaluate the potential of carvacrol as a sustainable antimicrobial alternative for disease control in aquaculture, with broader implications for human and environmental health.

## 2 Materials and methods

### 2.1 Bacterial strains and reagents

*Vibrio campbellii* strain BB120 (= ATCC BAA-1116), stored at 40% glycerol at -80 °C, was first grown in Luria Bertani (LB) agar (Difco Laboratories, Detroit, MI, USA) prepared by supplementing 35 g L^-1^ of Instant Ocean (LB_35_). A single colony was inoculated into LB35 broth (Difco Laboratories, Detroit, MI, USA), incubated overnight at 28 °C under constant agitation (120 rpm). Bacterial cell numbers were determined spectrophotometrically at an optical density (OD) of 600 nm, according to the McFarland standard (BioMerieux, Marcy L’Etoile, France), with an OD of 1.000 corresponding to 1.2 x 10^9^ cells mL^-1^ (28).

Carvacrol (98% pure) was purchased from Sigma-Aldrich (Diegem, Belgium). Carvacrol was freshly prepared in sterile seawater as an aqueous dispersion immediately before each test. Briefly, a known amount of the required volume of carvacrol was added to water to obtain a final concentration of 13.3 mM, followed by vigorous vortexing (2 min) and sonication in a water-bath sonicator (5 min) until a visually homogeneous dispersion was obtained. The stock solution was freshly prepared for each experiment, and the solution was re-vortexed briefly before use.

### 2.2 Axenic hatching of *Artemia* larvae

Axenic *Artemia* were obtained by decapsulation and hatching according to the method described by Marques et al. (29). Briefly, 1.5 g of high-quality hatching cysts of *Artemia franciscana* (EG^®^ Type; INVE Aquaculture, Belgium) were hydrated in 89 ml of distilled water for 1 h. Afterwards, 3.3 ml of NaOH (32%; w/v) and 50 ml of NaOCl (50% available chlorine) were added to the hydrated cyst suspension to facilitate decapsulation, with filtered (0.22 µm) aeration provided during the reaction for 2 min. The process was stopped by adding 50 ml of Na_2_S_2_O_3_ (10 g L^−1^). The aeration was then terminated, and the embryos were washed with filtered (0.22 µm) and sterile (moist heat at 121 °C for 20 min) artificial seawater containing 35 g L^-1^ of Instant Ocean synthetic sea salt (Aquarium Systems, Sarrebourg, France). The embryos were re-suspended in 500 ml of filtered, autoclaved seawater and hatched for at least 28 h at 28 °C with constant illumination of approximately 2000 lux. Air was bubbled through the suspension by a sterile tube extending to the bottom of the hatching vessel to keep all the cysts in continuous motion. All these manipulations were performed under a laminar flow cabinet to maintain the sterility of the embryos and the emerging nauplii. After 28h of incubation, the emerged nauplii that reached the instar II stage (the stage at which they can ingest bacteria) were used for the subsequent assays.

### 2.3 Standardized challenge test

The effect of carvacrol on the virulence of *V. campbellii* strain was determined in a standardised challenge test. Groups of 30 nauplii were transferred to sterile 40 mL glass tubes that contained 30 mL of filtered and autoclaved seawater. *V. campbellii* was incubated overnight, and the culture was washed before inoculation into the *Artemia* culture water at 10^7^ CFU mL^-1^. Carvacrol at different concentrations (0, 3.3, 6.6, 33.3, 66.6, 332.8 µM) was added directly into the rearing water together with the pathogen. *Artemia* nauplii without a challenge were used as control groups. Finally, the tubes were incubated on a rotor (4 min^−1^) at 28 °C. Survival of the nauplii was recorded after 48 h of challenge. Each treatment was carried out in five replicates, and each experiment was repeated at least once to verify the reproducibility. In each test, the sterility of the control groups was checked at the end of the challenge by inoculating 1 mL of rearing water into 9 ml of LB_35_ and incubating for 2 days at 28 °C (28). Results obtained with non-sterile nauplii were discarded.

### 2.4 Carvacrol toxicity assay

The potential toxic effect of carvacrol was determined in axenic *Artemia* nauplii as previously described (27) with some modifications. Hatched *Artemia* nauplii at developmental stage II were collected, a group of 30 nauplii was counted and thereafter transferred to 40-ml sterile glass bottles containing 30-ml sterile seawater. Different doses of carvacrol (0, 3.3, 6.6, 33.3, 66.6, 332.8 µM) were added to the *Artemia* rearing water as described in the challenge test above. *Artemia* nauplii that did not receive compound treatment served as a control. Five replicate *Artemia* cultures were maintained for each treatment and control. The toxicity of the compound was determined after 48 h of exposure to the compound by scoring the number of survivors as previously described (27).

### 2.5 Expression of immune-related genes by quantitative reverse transcription PCR

Expression of immune-related genes, including *β-actin* (reference), *proPO* (encoding prophenoloxidase), *hsp70* (heat shock protein 70), *tgase* (encoding transglutaminase), *ftn* (encoding ferritin), was analyzed by RT-qPCR using methods according to Baruah (27). Gene-specific primers shown in **Table 1** (designed by cross-exon strategy and the software PRIMER version 5.0) were described by previous studies (30, 31). After 28 h of incubation at 28 °C, swimming nauplii were collected, counted using a standard volumetric method, and then transferred to 500 mL sterile glass bottles. The nauplii were challenged with *V. campbellii* at 10^7^ cells mL^-1^, with or without carvacrol in an optimized dose. Each treatment was carried out in triplicate. Samples containing 0.1 g of live nauplii were harvested from all treatments at 6, 12, and 24 h post challenge, rinsed in cold distilled water, immediately frozen in liquid nitrogen, and stored at -80 °C for RNA extraction.

**Table 1:** Specific primers used for reverse transcriptase PCR of immune-related genes.

Gene	Primer sequences (5’-3’)
*β-actin*	GGTCGTGACTTGACGGACTATCT AGCGGTTGCCATTTCTTGTT
*hsp70*	CGATAAAGGCCGTCTCTCCA CAGCTTCAGGTAACTTGTCCTTG
*proPO*	CGCTGGCATAAGCACATCGATG GTCATTTCTCACTGTGAAACG
*ftn*	TCCAAGGCTTATCCGATGAACA ATGACCAAGTGAGTGCTTCTCT
*tgase*	TCTCTCCGTGTCTCTCCAAAAG CCCCACAAGAAGCATCTGAAG

Total RNA was extracted from *Artemia* nauplii using the RNeasy Plus Mini Kit (Qiagen, Germany) according to the manufacturer’s instructions and treated with RNase-free DNase to remove DNA contamination, after which the RNA was quantified spectrophotometrically by NanoDrop ™ 2000 (Thermo Scientific, USA). First-strand cDNA was synthesized from 1 μg of total RNA using the RevertAid™ H minus First-strand cDNA synthesis kit (Fermentas GmbH, Germany) according to the manufacturer’s instructions. The RT-qPCR assay was performed on Step One Plus Real-Time System (Applied Biosystems, USA) using Maxima SYBR Green/ROX qPCR Master Mix (2X) (Thermo Scientific, USA) in a total volume of 20 μL, containing 10 μL of 2X SYBR green master mix, 1 μL of forward and reverse primers (10 nM), 1 μL of template cDNA (10 ng), and 7 μL of nuclease-free water. The thermal cycling consisted of an initial denaturation at 95 °C for 10 min, followed by 40 cycles of denaturation at 95 °C for 15 s and primer annealing and elongation at 60 °C for 1 min. Dissociation curve analysis was performed to check for the amplification of untargeted fragments. The RT-qPCR assay was performed on three technical and three biological replicates for each sample. The Ct values of the target genes were normalized against the Ct value of the reference *Artemia* actin gene, which was used as an internal control. The fold changes relative to actin were calculated using the 2^-ΔΔCt^ method (32). Briefly, the Ct value of the target gene was first normalized to the Ct value of the reference gene to obtain ΔCt. The ΔCt of each treated sample was then compared with that of the control group to obtain ΔΔCt. For each time point, the control group was used as the calibrator and its expression level was set to 1.0. RT-qPCR data of three independent experiments are shown as the mean ± SE (standard error).

### 2.6 Impact of carvacrol on the growth of *V. campbellii* BB120

*Vibrio campbellii* BB120 was grown overnight in LB_35_ medium at 28 °C on a shaker. Subsequently, the suspension was diluted 1:50 (v/v) in LB medium supplemented with the most effective carvacrol dose. LB medium without supplement was used as a control. After inoculation, the suspensions were incubated at 28 °C under constant shaking. Growth was monitored by measuring the optical density (OD_600_) during 24 h. Each treatment was performed in triplicate.

### 2.7 Biofilm formation assay

Biofilm formation was quantified using crystal violet staining as described by Zheng et al. (33). In brief, overnight cultures of *V. campbellii* strain were diluted to an OD_600_ of 0.1 in LB_35_, and 200 mL aliquots were pipetted into the wells of a 96-well plate. Then the plate was incubated without agitation for 24 h at 28 °C to allow the bacteria to adhere and grow. After that, the planktonic cells were removed, and the wells were washed three times with 300 mL sterile physiological saline. The remaining attached bacteria were fixed with 200 mL of methanol per well for at least 20 min, after which the methanol was removed, and the plates were air-dried. Subsequently, biofilms were stained with 200 mL of a 0.1% crystal violet solution (Sigma- Aldrich, Belgium) per well for 15 min. Excess stain was rinsed off, and after the plates were air dried, the dye bound to the adherent cells was resolubilized with 200 mL of 95% ethanol per well, and absorbance was measured at 570 nm. Sterile medium served as a negative control.

### 2.8 Virulence factor assays

All enzymatic assays were performed according to Natrah et al. (34) with some modifications. In all assays, *V. campbellii* BB120 was cultured overnight in LB_35_ broth, and 5 µL aliquots (OD_600_ = 1) were spotted in the center of the test plates. Carvacrol was added to the autoclaved agar immediately before pouring the plates. All assays were performed in triplicate.

Hemolytic assay plates were prepared by supplementing LB_35_ agar with 5% defibrinated sheep blood (Oxoid, Basingstoke, Hampshire, UK). After the addition of bacterial suspension on the surface of the agar plates, the latter were incubated upright at 28 °C. The clearing zones were observed and measured after incubation for 48 h. The caseinase plates were prepared by mixing double-strength LB_35_ agar with skim milk powder suspension (4%; autoclaved separately at 121 °C for 5 min). Clearing zones around the bacterial colonies were observed and measured after 48 h of incubation.

Lipase and phospholipase activities were assessed using LB_35_ plates containing 1% Tween 80 (Sigma-Aldrich, Belgium) and 1% egg yolk emulsion (Sigma-Aldrich, Belgium), respectively. The development of opalescent zones around the colonies was observed, and the diameters of the zones were recorded after 2–4 days of incubation at 28 °C.

### 2.9 Statistical analysis

All statistical analyses were performed using statistical software SPSS version 20.0. The percentage-based survival data were arcsine transformed before analysis to meet the assumptions of normality and homoscedasticity of variances using Shapiro-wilk and Levene´s tests, respectively. When these assumptions were met, the transformed data were subjected to one-way analysis of variance (ANOVA) followed by Tukey’s *post hoc* test. The RT-qPCR data were also tested for normality and equal variance as described above before performing the ANOVA. In cases where the variances were unequal, the survival and RT-qPCR data were analyzed using the non-parametric Kruskal-Wallis test.

Statistical comparisons of the gene expression data were performed separately for each time point. Within a given time point, the expression of the target gene in the control group was set at 1.0, and all other data points were normalized accordingly using the equation of the 2^-ΔΔCT^ method. Significant differences in growth and virulence-related data were determined by Student’s *t*-test. Significance level was set at *P* < 0.05.

## 3 Results

### 3.1 Carvacrol protects *Artemia* nauplii from *V. campbellii* infection

**Figure 1:**
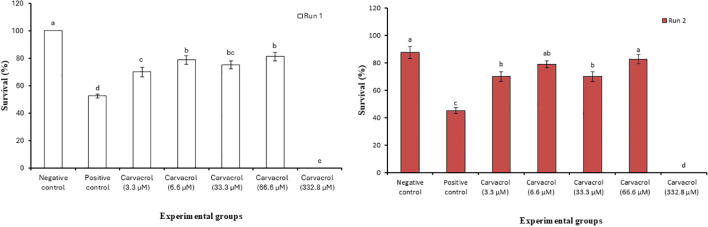
Impact of carvacrol on the survival of *Artemia nauplii* after 48h challenge with *V. campbelli* BB120. Error bars represent the standard error of five replicates. Different letters indicate significant differences (one-way ANOVA with Tukey’s *post hoc* test; *P* < 0.05). “Run 1” and “Run 2” represent two independent repetitions of the same challenge experiment, conducted to confirm the reproducibility of the results.

We first performed a dose-response study to determine the optimal dosage of carvacrol that could protect *Artemia nauplii* against *V. campbellii*. Following this, we challenged the nauplii with *V. campbelii*. The survival of challenged *Artemia nauplii* treated with carvacrol exhibited a significant increase compared to the control group at all concentrations tested, except for 332.8 µM (**Figure 1**; *P* < 0.05). The best protection was recorded in the group exposed to 66.6 µM of carvacrol (1.6-fold higher than the control in run 1; 1.8-fold higher in run 2), followed by 6.6 µM of carvacrol (1.5-fold higher in run 1; 1.8-fold higher in run 2). No significant differences in survival were observed among the groups exposed to 6.6, 33.3, and 66.6 µM carvacrol in Run 1 (*P* > 0.05), and a comparable trend was observed in Run 2. Notably, in Run 2, the groups treated with 6.6 µM and 66.6 µM carvacrol showed complete protection, as their survival rates did not differ significantly from those of the negative control (*P* > 0.05).

### 3.2 Toxicity of carvacrol towards *Artemia*

**Figure 2:**
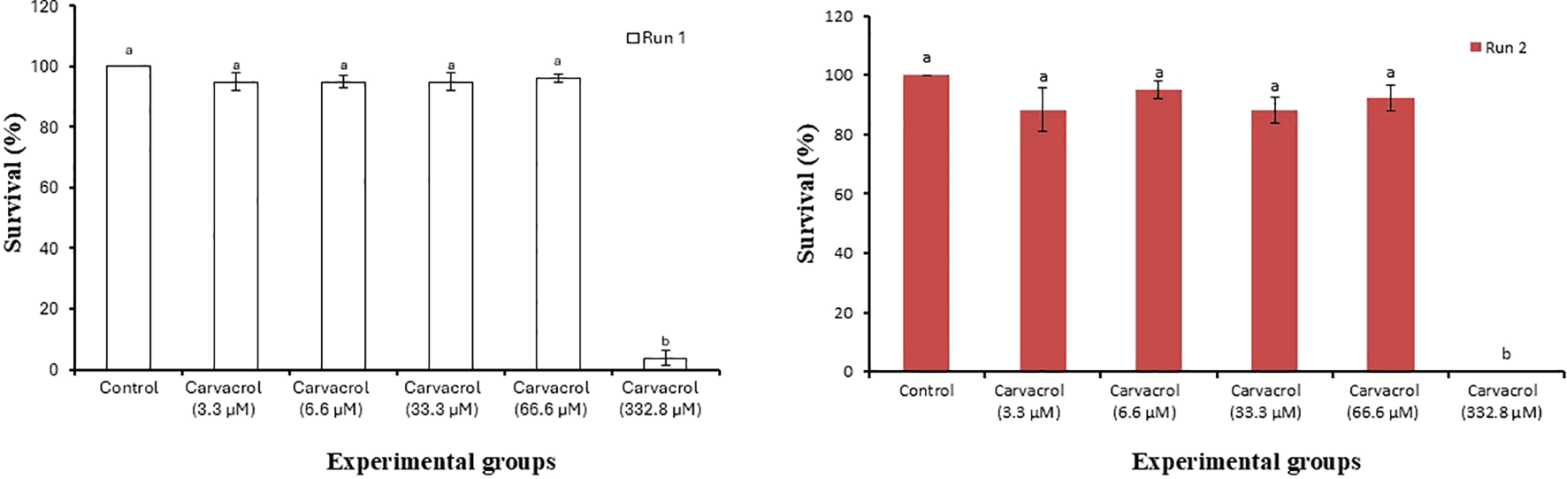
Toxicity of carvacrol against *Artemia* nauplii. Error bars represent the standard error of five replicates. Different letters indicate significant differences (one-way ANOVA with Tukey’s *post hoc* test; *P* < 0.05). “Run 1” and “Run 2” represent two independent repetitions of the same challenge experiment, conducted to confirm the reproducibility of the results.

Since a 100% mortality was observed in the presence of 332.8 µM carvacrol, we next evaluated the toxicity of carvacrol to *Artemia* nauplii. As shown in **Figure 2**, carvacrol did not induce mortality in *Artemia* nauplii for concentrations up to 66.6 µM. However, the survival of the nauplii significantly decreased at 332.8 µM (*P* < 0.05) in both the runs (Run 1 and Run 2) conducted independently to check the reproducibility of the results. Based on this result, together with the data from the challenge test, a dosage of 6.6 µM was selected for further experiments.

### 3.3 Determination of the impact of carvacrol on the growth of *V. campbelii*

**Figure 3:**
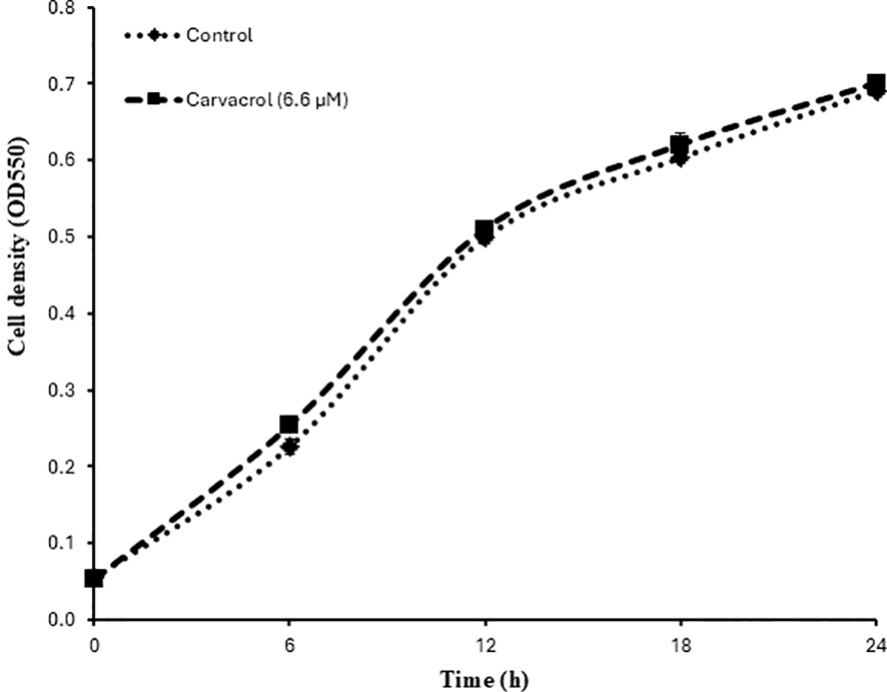
Impact of carvacrol on the growth of *V. campbellii* BB120 in LB medium containing 35 g/L of synthetic sea salt. Error bars represent the standard error of 3 replicate cultures.

To investigate whether the protection was due to the antibacterial activity of carvacrol on the pathogen, we determined the impact of carvacrol on the growth of *V. campbelii* in LB_35_ medium at an optimal concentration (6.6 µM). According to **Figure 3**, carvacrol at this concentration showed no effect on the growth of the pathogen.

### 3.4 Determination of the impact of carvacrol on virulence factors production in *V. campbellii*

**Figure 4:**
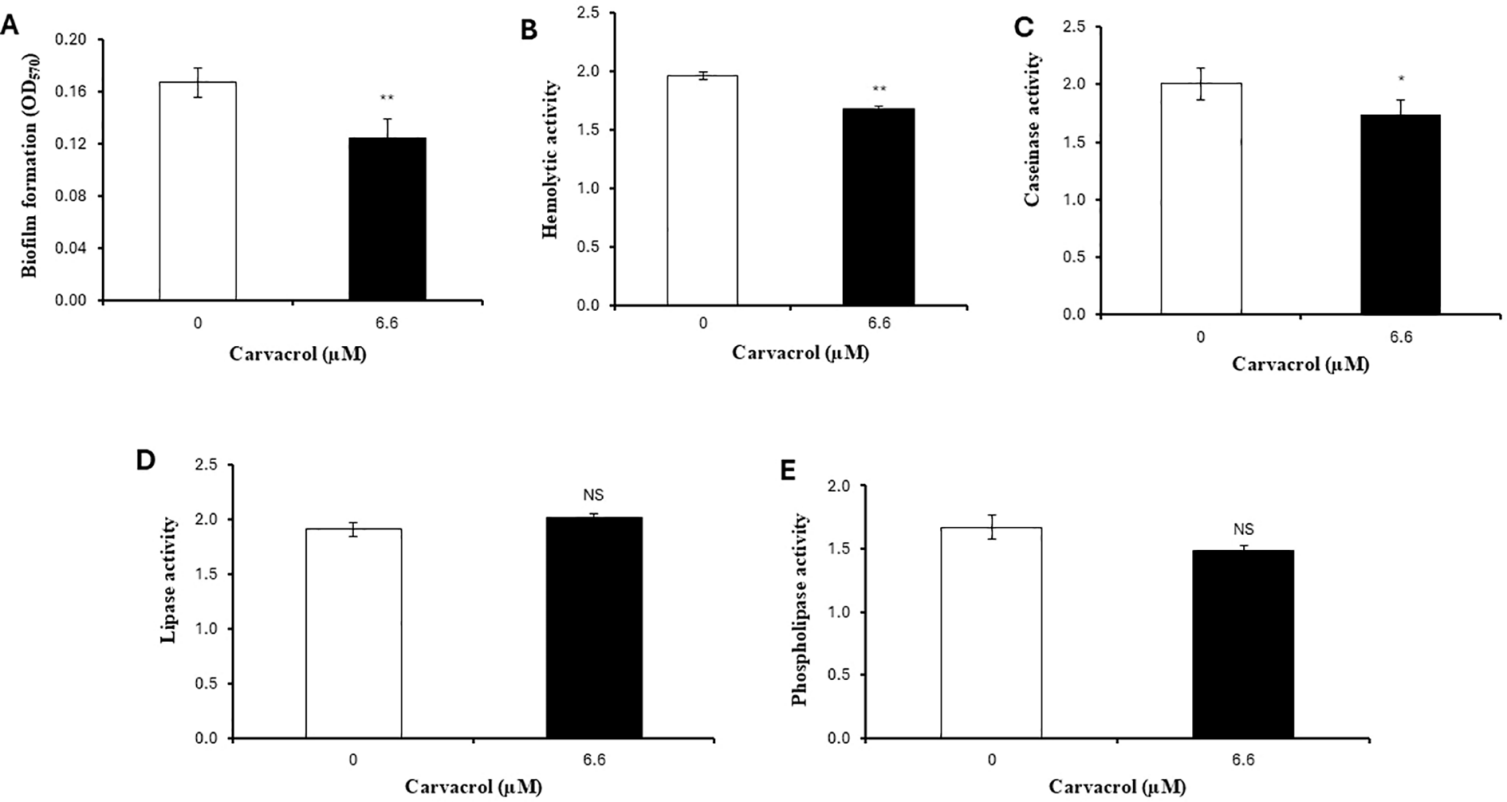
Impact of carvacrol on **(A)** biofilm formation, **(B)** hemolytic activity, **(C)** caseinase activity, **(D)** lipase activity, and **(E)** phospholipase activity of *V. campbellii* BB120. The activities of different virulence-related enzymes were expressed as the ratio between the activity zone and colony diameter. Error bars represent the standard error of three replicates. Asterisks indicate a significant difference when compared with non-treated BB120 (independent samples *t*-test; NS, not significant; *P < 0.1, **P < 0.01).

We further investigated the impact of carvacrol on phenotypes that have been shown to contribute to the virulence of *V. campbellii*. Although carvacrol significantly decreased the biofilm formation, hemolytic and caseinase activities (**Figures 4A–C**), the differences were relatively modest (< 1.5-fold). No significant effect of the compound was observed in the lipase and phospholipase activities (**Figures 4D, E**; *P* > 0.05).

### 3.5 *In vivo* determination of the impact of carvacrol on the expression of innate immune-related genes in *Artemia*

To further assess the impact of carvacrol on the host, we investigated whether the observed differences in *Artemia* survival associated with carvacrol treatment were linked to changes in innate immune-related gene expression. Nauplii from both the control and carvacrol-treated groups were sampled at 6, 12, and 24 h post-infection. These timepoints were selected to capture early immune signaling and response events that precede measurable mortality and final survival outcomes.

#### 3.5.1 Impact of carvacrol on *hsp70* expression

**Figure 5:**
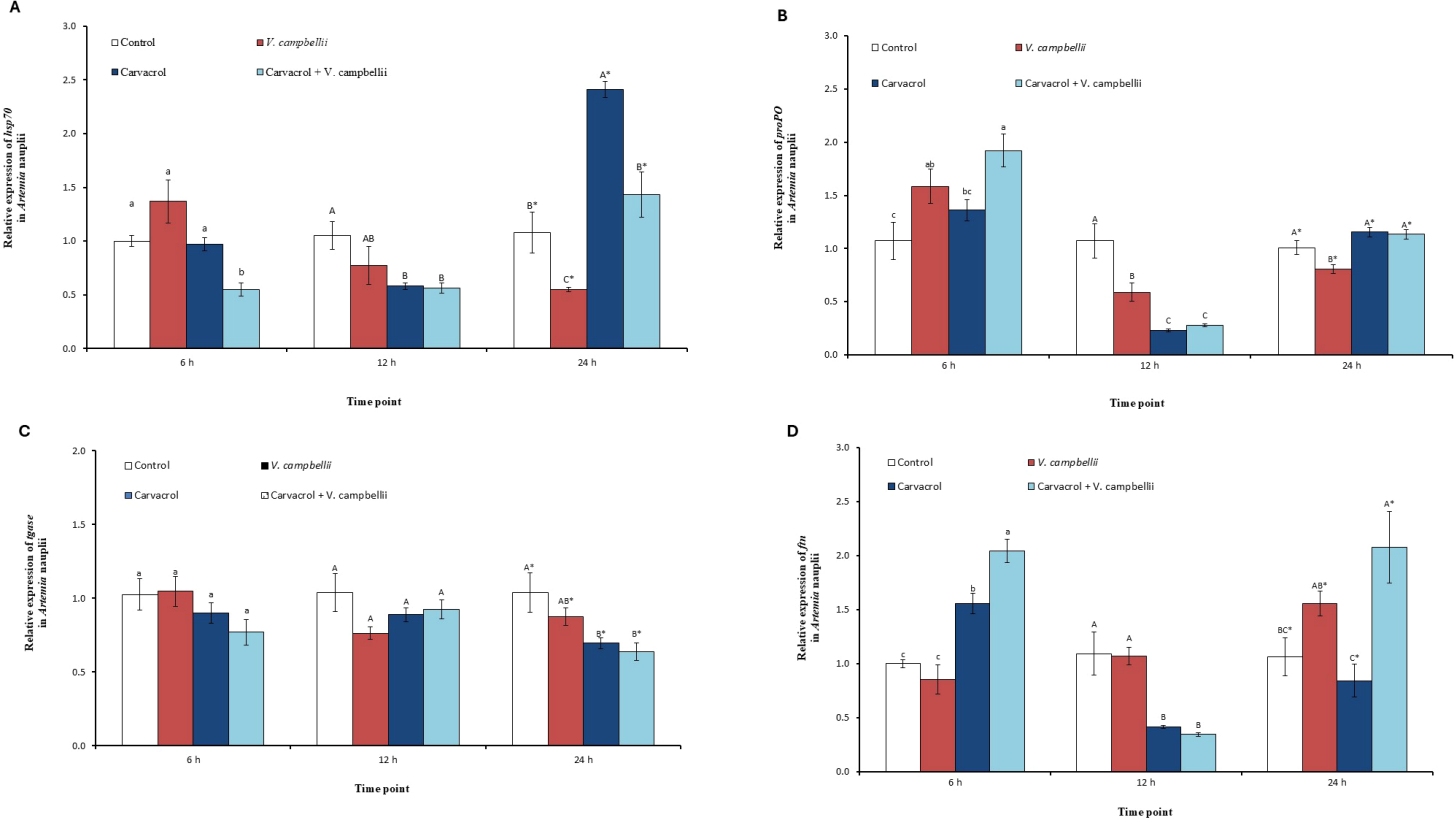
Impact of carvacrol (6.6 µM) treatment on the expression of **(A)** *hsp70*, **(B)** *proPO*, **(C)** *ftn* and **(D)** *tgase* genes in *Artemia* nauplii at 6, 12 and 24 h after challenge with *Vibrio campbellii* BB120. Error bars represent the standard errors of three biological replicates. Within each time point, bars sharing the same letters are not significantly different, whereas different letters indicate significant differences (one-way ANOVA with Tukey’s *post hoc* test; *P* < 0.05). Lowercase letters refer to 6 h, uppercase letters to 12 h, and asterisk letters to 24 h post-challenge time point.

At 6 h post-treatment, *hsp70* expression remained largely comparable to the control across most groups (**Figure 5A**). Untreated *Artemia* challenged with *V. campbellii* showed a mild increase in *hsp70* transcription (approximately 1.4-fold) relative to the control. However, this difference was not statistically significant (*P* > 0.05). Similarly, carvacrol treatment alone yielded expression levels comparable to those of the control. In contrast, carvacrol-treated *Artemia* challenged with *V. campbellii* (carvacrol + BB120) exhibited a significant downregulation of *hsp70* compared with the control (by 0.55-fold; *P* < 0.05) and all other treatments. At 12 h post-treatment, a general decline in *hsp70* expression was observed in all the treated groups. *Artemia* exposed to *V. campbellii* alone showed a moderate reduction (by 0.75-fold), although this decrease was not significant (*P* > 0.05). Meanwhile, carvacrol treatment, both in the absence and presence of bacterial challenge, resulted in a significant reduction of *hsp70* expression, with levels decreasing to approximately 0.60-fold (*P* < 0.05). By 24 h post-treatment, the expression pattern shifted substantially. *Artemia* treated with carvacrol alone displayed a pronounced and significant induction of *hsp70*, reaching the highest value among all groups (2.4-fold; *P* < 0.05). However, carvacrol-treated *Artemia* challenged with *Vibrio* showed only a moderate increase in *hsp70* expression (1.4-fold; *P* > 0.05) compared with the control, but this level was significantly lower than that observed with carvacrol treatment alone. Conversely, *Artemia* challenged with *V. campbellii* in the absence of carvacrol exhibited the lowest *hsp70* expression (0.55-fold), suggesting sustained repression of *hsp70* under bacterial challenge without carvacrol supplementation.

#### 3.5.2 Impact of carvacrol on *proPO* expression

At 6 h post-treatment, *Artemia* exposed to *V. campbellii* showed a significant upregulation of *proPO* (1.6-fold; *P* < 0.05), while *Artemia* treated with carvacrol alone did not exhibit a significant increase in the expression level (**Figure 5B**). The highest *proPO* transcription at this time point was observed in the carvacrol-treated group that was challenged (1.9-fold), which was significantly higher than the control group (*P* < 0.05). At 12 h post-treatment, the *proPO* expression level declined significantly in all the treated groups (*P* < 0.05). The *V. campbellii*–challenged group showed a significant reduction in expression (0.6-fold) compared with the control (*P* < 0.05). A more pronounced decline was recorded in the carvacrol-treated groups, regardless of bacterial challenge, where *proPO* levels dropped to approximately 0.25-fold, representing the lowest expression values at this time point (*P* < 0.05). By 24 h post-challenge, the *proPO* level recovered in most treatments. Both *Artemia* treated with carvacrol alone, and those treated with carvacrol and simultaneously challenged with *V. campbellii* showed expression levels similar to the control (1.1–1.2-fold; *P* > 0.05), suggesting a return to baseline transcription. In contrast, *Artemia* challenged with *V. campbellii* alone maintained a significantly reduced expression level (0.8-fold) compared with the control and carvacrol-treated groups (*P* < 0.05).

#### 3.5.3 Impact of carvacrol on *tgase* expression

At 6 h post-treatment, the relative expression of *tgase* in *Artemia* nauplii remained close to the control level in all groups, with no significant differences among treatments (*P* > 0.05; **Figure 5C**). A similar trend was observed at 12 h post-treatment. At 24 h, a clearer treatment-related pattern was observed. The expression level of *tgase* was reduced in the *V. campbellii* group compared with the control, while nauplii treated with carvacrol, both in the presence and absence of bacterial challenge, showed the lowest expression levels. In particular, the carvacrol-treated groups exhibited a significant downregulation of *tgase* expression (approximately 0.7-fold) relative to the control (*P* < 0.05).

#### 3.5.4 Impact of carvacrol on *ftn* expression

At 6 h post-treatment, the relative expression of *ftn* in *Artemia* nauplii increased significantly in the carvacrol-treated groups (**Figure 5D**; *P* < 0.05). While the control and *V. campbellii*-challenged groups remained close to baseline, carvacrol alone significantly upregulated *ftn* expression (1.6-fold; *P* < 0.05), and the carvacrol-treated *Artemia* in the presence of *V. campbellii* challenged showed the highest induction (2.0-fold; *P* < 0.05). At 12 h, the expression level of *ftn* decreased in the carvacrol-treated nauplii. Both carvacrol-treated groups, regardless of *Vibrio* challenge, exhibited a significant downregulation (0.35–0.45-fold) compared to the control (*P* < 0.05), whereas the control and *V. campbellii*-challenged groups remained around baseline levels. By 24 h post-treatment, the expression of *ftn* increased again, particularly in the challenged groups. *Artemia* exposed to *V. campbellii* alone showed a significant elevation in the *ftn* expression level (*P* < 0.05), while carvacrol alone remained at a similar level to the control. Notably, carvacrol-treated *Artemia* and simultaneously challenged with *V. campbellii* resulted in the strongest upregulation of *ftn*, reaching approximately 2.1-fold, and was significantly higher than the other groups (*P* < 0.05).

## 4 Discussion

Multi-drug resistance is spreading rapidly in aquaculture, rendering traditional antibiotic treatments ineffective and posing a threat to public health. Antimicrobial resistance of over 11,200 different bacterial isolates from farmed aquatic animals and their environment was obtained from the literature (6). In addition, aquaculture products may serve as a source of antibiotic resistance genes that can be transferred to human microbiota, posing a One Health risk. Therefore, effective novel alternative approaches to combat bacterial infection are urgently needed, in line with the Food and Agriculture Organization’s Action Plan on antimicrobial resistance (35).

Before the age of antibiotic exploration, the utilization of traditional herbal extracts as curative agents for certain diseases was prevalent across the world. Based on extensive literature evidence, the major constituent of the essential oil fraction of oregano, carvacrol, was selected in this study to evaluate its bioactive properties on the aquaculture pathogen *V. campbellii*. Here, we report for the first time on the anti-virulence property of carvacrol against *V. campbellii*. Carvacrol significantly improved the survival of challenged *Artemia* nauplii compared with the control, except at the highest concentration tested, confirming earlier findings that carvacrol pre-treatment enhances resistance to infection (27). To assess host safety, we also evaluated potential toxicity and found no toxic effects in *Artemia* nauplii at concentrations below 332.8 µM.

In general, the infectious cycle of pathogenic bacteria consists of several stages, such as pathogen entry, establishment and proliferation, evasion of host defence response, infliction of damage, and so on. Each of these stages involves the production of different virulence factors (36). To further determine the underlying mechanisms of how carvacrol could protect *Artemia* from bacterial infection, several important virulence factors were examined. The first virulence factor we tested is biofilm formation, which is considered an essential factor in the persistence of pathogenic bacteria in hostile environments (37, 38). Biofilm is a community of microbial cells that are attached to a surface and/or to each other, and enclosed in a self-produced extracellular polymeric substance matrix, which is composed of polysaccharides, lipids, proteins, and extracellular DNA (39). Bacteria in the biofilm state usually exhibit higher resistance to physical/chemical disinfectants, antibiotic treatment and the host immune system than planktonic bacteria, making biofilm-forming *Vibrio* species a major threat to the aquaculture industry (40, 41). In *V. campbellii*, biofilm production has been reported to be involved in virulence under the regulation of QS (42). Therefore, it is desirable to select antimicrobial agents with the ability to inhibit or eliminate biofilm formation. In the present study, we found that biofilm level was significantly reduced in the presence of carvacrol at 6.6 µM. These findings are in line with previous studies documenting biofilm inhibitory activity of carvacrol in different bacteria (43, 44).

Hemolysins and caseinase are the crucial extracellular virulence factors produced by *V. campbellii*. These enzymes play a critical role in damaging host tissues, enabling the pathogen to acquire nutrients and spread within the host (36). We observed that carvacrol could only slightly inhibit the hemolytic and caseinase activity of *V. campbellii* at the tested concentration. As significant virulence factors of pathogens, the effect of carvacrol on hemolytic and caseinase activity has also been explored intensively by other researchers. It has been reported that carvacrol markedly decreased the protease production and hemolytic activity in a dose-dependent pattern in *Areomonas hydrophila* (45). In another work of Kim et al. (46), carvacrol did not change the protease and lipase activities but reduced the hemolytic and nuclease activities in *Staphylococcus aureus*. Moreover, sub-lethal concentrations of carvacrol reduced chitinase activity in *Chromobacterium violaceum* (44). Based on these results, the impact of carvacrol on hemolytic and caseinase activities is species dependent.

Antimicrobial alternatives that combine anti-virulence activity with immune-stimulatory effects can be particularly useful for disease control, as they act on both the pathogen and the host. By weakening pathogen virulence while improving host defence capacity, such dual-action strategies may provide superior protection under aquaculture conditions, where animals often encounter changing conditions, such as fluctuations in temperature, water quality and microbial communities and handling stress. These stressors can compromise immune function and increase susceptibility to opportunistic infections. In a previous study, we demonstrated that prior exposure to carvacrol can induce Hsp70 in *Artemia* nauplii, which improved resistance towards subsequent critical stressors, including lethal heat exposure and bacterial infection (27). Hsp70 is a highly conserved stress-responsive protein and is widely used as a biomarker of cellular stress and immune activation (47). Evidence suggests that Hsp70 can strengthen immune responsiveness in various animals, including *Artemia*, against bacterial diseases by production of immune effector molecules, such as proPO, transglutaminase, ferritin, and antimicrobial peptides (31, 48–50).

To better understand whether the improved survival in carvacrol-treated nauplii was linked to host defence responses, we examined the transcriptional response of *hsp70* and other key innate immune genes *proPO*, *ftn* and *tgase*, which encode for the immune effector proteins phenoloxidase, ferritin and transglutaminase, respectively. Our results indicate that carvacrol appears to reduce infection-associated stress during the early stage of infection, as evidenced by a significant reduction of *hsp70* in the carvacrol-treated *Artemia* challenged with *Vibrio*, compared with the slight increase observed in the untreated challenged group. This may suggest that carvacrol reduces the burden of infection on the host, possibly by reducing bacterial virulence as observed in our study. At 12 h post-challenge, the pattern shifts noticeably. Carvacrol alone strongly upregulated *hsp70*, suggesting that it may also activate a delayed stress-adaptive response. When carvacrol was combined with infection, this increase was less pronounced, indicating that bacterial challenge can influence the magnitude of the adaptive response. This divergence highlights that carvacrol protects *Artemia*, possibly by lowering early stress during infection while also supporting later stress adaptation.

The *proPO*, *tgase* and *ftn* genes are major components of the crustacean innate immunity (51, 52). The proPO system is essential for defence against pathogens, as activation of phenoloxidase contributes to melanization reactions, wound healing, encapsulation and pathogen killing (52). Ferritin (*ftn*) is a key component of iron homeostasis and nutritional immunity, restricting free iron availability that can otherwise support bacterial growth. Transglutaminase (*tgase*) contributes to defence by supporting hemolymph coagulation, which can help prevent the loss of hemolymph through injuries in the exoskeleton, and the subsequent entry and proliferation of microbes throughout the body (53, 54). Our results suggested that carvacrol influences these immune pathways in a time-dependent manner during bacterial challenge. During the early infection stage (i.e., 6 h post-challenge), carvacrol appeared to stimulate a state of heightened immune response, as shown by increased expression of *proPO* and *ftn* in treated and challenged *Artemia*. This suggests that carvacrol may help the host respond more effectively at the start of infection through melanization-related defense and iron sequestration, potentially limiting bacterial growth and reducing host damage. At 24 h, the strong induction of *ftn* in challenged carvacrol-treated nauplii further supports the suggestion that an iron-withholding defence may contribute to improved survival. In contrast, the observation that the expression of *tgase* remained stable at 6 h and 12 h across treatments suggests that this defence pathway may not be strongly involved during the early response under the present experimental conditions. However, at 24 h, *tgase* was significantly downregulated in both carvacrol-treated groups, similar to the declining trend observed in the *Artemia* challenged with *Vibrio* only. This reduction may suggest reduced clotting-related immune activity due to improved control of infection in the carvacrol-treated challenged group, though the functional implications of this transcriptional suppression require further investigation at the protein level. Our findings are in agreement with earlier studies in gnotobiotic *Artemia*, showing that bioactive compounds (e.g. carvacrol, pyrogallol, butyrate) improve the resistance of the host towards *Vibrio* infection by modulating innate immune gene expression in a time-dependent and stochastic pattern, rather than by sustained upregulation of the genes. Similar fluctuating transcriptional patterns involving *hsp70, proPO*, and *tgase*, together with enhanced survival after *V. parahaemolyticus* challenge, have been reported in penaeid shrimp treated with immunostimulatory compounds (55), reinforcing that enhanced protection does not necessarily require sustained upregulation of all immune markers, but rather an effective and well-regulated immune response over time.

## 5 Conclusion

In essence, our results suggest that carvacrol is a promising natural antimicrobial against *V. campbellii*. Carvacrol significantly improved the survival of gnotobiotic *Artemia* during infection while showing low toxicity at relevant concentrations. Mechanistically, the protective outcome was associated with reduced pathogen virulence traits and time-dependent regulation of host stress and immune genes. The effects of carvacrol on virulence factor production were assessed using *in vitro* approaches in this study, and the infection experiments were performed in a gnotobiotic model to allow controlled evaluation of host-pathogen interactions. While this system provides a valuable framework to elucidate mechanistic effects without interference from background microbiota, it represents a simplified environment compared to natural aquaculture systems. Therefore, future *in vivo* virulence studies under more complex microbial and environmental conditions are warranted to validate whether the observed protective and anti-virulence effects are maintained under realistic farming conditions. Taken together and given its approval for use as a safe food-grade component, carvacrol holds strong translational potential as a functional antimicrobial strategy to support disease control and health management in aquaculture, warranting further evaluation under realistic farming conditions.

## Data Availability

The raw data supporting the conclusions of this article will be made available by the authors, without undue reservation.

## References

[1] Fisheries FA . The state of world fisheries and aquaculture 2024 blue transformation in action. doi: 10.4060/cd0683en

[2] Maldonado-Miranda JJ Castillo-Pérez LJ Ponce-Hernández A Carranza-Álvarez C . Summary of economic losses due to bacterial pathogens in aquaculture industry. In: Bacterial fish diseases. Cambridge, MA, USA: Academic Press (2022). p. 399–417. doi: 10.1016/B978-0-323-85624-9.00023-3, PMID:

[3] Darshanee Ruwandeepika HA Sanjeewa Prasad Jayaweera T Paban Bhowmick P Karunasagar I Bossier P Defoirdt T . Pathogenesis, virulence factors and virulence regulation of vibrios belonging to the Harveyi clade. Rev Aquacult. (2012) 4:59–74. doi: 10.1111/j.1753-5131.2012.01061.x. PMID: 41834780

[4] Vicente A Taengphu S Hung AL Mora CM Dong HT Senapin S . Detection of Vibrio campbellii and V. parahaemolyticus carrying full-length pirABVp but only V. campbellii produces PirVp toxins. Aquaculture. (2020) 519:734708. doi: 10.1016/j.aquaculture.2019.734708. PMID: 41847267

[5] Baker-Austin C Trinanes JA Taylor NG Hartnell R Siitonen A Martinez-Urtaza J . Emerging Vibrio risk at high latitudes in response to ocean warming. Nat Clim Change. (2013) 3:73–7. doi: 10.1038/nclimate1628. PMID: 41839915

[6] Reverter M Sarter S Caruso D Avarre JC Combe M Pepey E . Aquaculture at the crossroads of global warming and antimicrobial resistance. Nat Commun. (2020) 11:1870. doi: 10.1038/s41467-020-15735-6. PMID: 32312964 PMC7170852

[7] Hundenborn J Thurig S Kommerell M Haag H Nolte O . Severe wound infection with Photobacterium damselae ssp. damselae and Vibrio harveyi, following a laceration injury in marine environment: a case report and review of the literature. Case Rep Med. (2013) 2013:610632. doi: 10.1155/2013/610632. PMID: 24171004 PMC3792539

[8] Huang J Zeng B Liu D Wu R Zhang J Liao B . Classification and structural insight into vibriolysin-like proteases of Vibrio pathogenicity. Microb Pathogen. (2018) 117:335–40. doi: 10.1016/j.micpath.2018.03.002. PMID: 29510206

[9] Thompson FL Gevers D Thompson CC Dawyndt P Naser S Hoste B . Phylogeny and molecular identification of vibrios on the basis of multilocus sequence analysis. Appl Environ Microbiol. (2005) 71:5107–15. doi: 10.1128/AEM.71.9.5107-5115.2005. PMID: 16151093 PMC1214639

[10] Defoirdt T Boon N Sorgeloos P Verstraete W Bossier P . Alternatives to antibiotics to control bacterial infections: luminescent vibriosis in aquaculture as an example. Trends Biotechnol. (2007) 25:472–9. doi: 10.1016/j.tibtech.2007.08.001. PMID: 17719667

[11] Watts JE Schreier HJ Lanska L Hale MS . The rising tide of antimicrobial resistance in aquaculture: sources, sinks and solutions. Mar Drugs. (2017) 15:158. doi: 10.3390/md15060158. PMID: 28587172 PMC5484108

[12] Tarin-Pello A Suay-Garcia B Perez-Gracia MT . Antibiotic resistant bacteria: current situation and treatment options to accelerate the development of a new antimicrobial arsenal. Expert Rev Anti-Infective Ther. (2022) 20:1095–108. doi: 10.1080/14787210.2022.2078308. PMID: 35576494

[13] Rahim AA Ahmadissa SM Muhamad LR Hama Soor TA . Antibiotic resistance: Current global issue and future challenges. Microbial Biosyst. (2021) 5:29–68. doi: 10.21608/mb.2021.55637.1029

[14] Lisin G Safiyev S Craker LE . (1997). Antimicrobial activity of some essential oils. Leuven, Belgium: International Society for Horticultural Science (ISHS), in: II WOCMAP Congress Medicinal and Aromatic Plants, Part 2: Pharmacognosy, Pharmacology, Phytomedicine, Toxicology. pp. 283–8. doi: 10.17660/ActaHortic.1999.501.45

[15] Tang X Chen S Wang L . Purification and identification of carvacrol from the root of Stellera chamaejasme and research on its insecticidal activity. Nat Prod Res. (2011) 25:320–5. doi: 10.1080/14786419.2010.532796. PMID: 21294044

[16] Fachini-Queiroz FC Kummer R Estevao-Silva CF Carvalho MD Cunha JM Grespan R . Effects of thymol and carvacrol, constituents of Thymus vulgaris L. essential oil, on the inflammatory response. Evidence‐Based Complementary Altern Med. (2012) 2012:657026. doi: 10.1155/2012/657026. PMID: 22919415 PMC3418667

[17] Stefanaki A Cook CM Lanaras T Kokkini S . The Oregano plants of Chios Island (Greece): Essential oils of Origanum onites L. growing wild in different habitats. Ind Crops Prod. (2016) 82:107–13. doi: 10.1016/j.indcrop.2015.11.086. PMID: 41847267

[18] Sharifi‐Rad M Varoni EM Iriti M Martorell M Setzer WN del Mar Contreras M . Carvacrol and human health: A comprehensive review. Phytother Res. (2018) 32:1675–87. doi: 10.1002/ptr.6103. PMID: 29744941

[19] Li P Chen X Aziz T Shami A Al-Asmari F Al-Joufi FA . The anti-biofilm and anti-virulence mechanisms of carvacrol against Listeria monocytogenes and the application in food systems. Food Biosci. (2025) 69:106950. doi: 10.1016/j.fbio.2025.106950. PMID: 41847267

[20] Mechmechani S Gharsallaoui A Fadel A El Omari K Khelissa S Hamze M . Microencapsulation of carvacrol as an efficient tool to fight Pseudomonas aeruginosa and Enterococcus faecalis biofilms. PloS One. (2022) 17:e0270200. doi: 10.1371/journal.pone.0270200. PMID: 35776742 PMC9249205

[21] Xu J Zhou F Ji BP Pei RS Xu N . The antibacterial mechanism of carvacrol and thymol against Escherichia coli. Lett Appl Microbiol. (2008) 47:174–9. doi: 10.1111/j.1472-765X.2008.02407.x. PMID: 19552781

[22] Nostro A Papalia T . Antimicrobial activity of carvacrol: current progress and future prospectives. Recent Patents Anti-Infective Drug Discov. (2012) 7:28–35. doi: 10.2174/157489112799829684. PMID: 22044355

[23] Siroli L Braschi G de Jong A Kok J Patrignani F Lanciotti R . Transcriptomic approach and membrane fatty acid analysis to study the response mechanisms of Escherichia coli to thyme essential oil, carvacrol, 2‐(E)‐hexanal and citral exposure. J Appl Microbiol. (2018) 125:1308–20. doi: 10.1111/jam.14048. PMID: 30028070

[24] Silva LN Zimmer KR Macedo AJ Trentin DS . Plant natural products targeting bacterial virulence factors. Chem Rev. (2016) 116:9162–236. doi: 10.1021/acs.chemrev.6b00184. PMID: 27437994

[25] Yan C Kuang W Jin L Wang R Niu L Xie C . Carvacrol protects mice against LPS-induced sepsis and attenuates inflammatory response in macrophages by modulating the ERK1/2 pathway. Sci Rep. (2023) 13:12809. doi: 10.17660/ActaHortic.1999.501.45. PMID: 37550359 PMC10406886

[26] Meijer MMY van den Brand H Niknafs S . In ovo delivery of carvacrol triggers expression of chemotactic factors, antimicrobial peptides and pro-inflammatory pathways in the yolk sac of broiler chicken embryos. J Anim Sci Biotechnol. (2025) 16:8. doi: 10.1186/s40104-024-01131-3. PMID: 39828746 PMC11742807

[27] Baruah K Norouzitallab P Phong HP Smagghe G Bossier P . Enhanced resistance against Vibrio harveyi infection by carvacrol and its association with the induction of heat shock protein 72 in gnotobiotic Artemia franciscana. Cell Stress Chaperones. (2017) 22:377–87. doi: 10.1007/s12192-017-0775-z. PMID: 28303510 PMC5425368

[28] Baruah K Cam DT Dierckens K Wille M Defoirdt T Sorgeloos P . *In vivo* effects of single or combined N-acyl homoserine lactone quorum sensing signals on the performance of Macrobrachium rosenbergii larvae. Aquaculture. (2009) 288:233–8. doi: 10.1016/j.aquaculture.2008.11.034. PMID: 41847267

[29] Marques A Dinh T Ioakeimidis C Huys G Swings J Verstraete W . Effects of bacteria on Artemia franciscana cultured in different gnotobiotic environments. Appl Environ Microbiol. (2005) 71:4307–17. doi: 10.1128/AEM.71.8.4307-4317.2005. PMID: 16085818 PMC1183358

[30] Baruah K Norouzitallab P Linayati L Sorgeloos P Bossier P . Reactive oxygen species generated by a heat shock protein (Hsp) inducing product contributes to Hsp70 production and Hsp70-mediated protective immunity in Artemia franciscana against pathogenic vibrios. Dev Comp Immunol. (2014) 46:470–9. doi: 10.1016/j.dci.2014.06.004. PMID: 24950414

[31] Norouzitallab P Baruah K Biswas P Vanrompay D Bossier P . Probing the phenomenon of trained immunity in invertebrates during a transgenerational study, using brine shrimp Artemia as a model system. Sci Rep. (2016) 6:21166. doi: 10.1038/srep21166. PMID: 26876951 PMC4753410

[32] Livak KJ Schmittgen TD . Analysis of relative gene expression data using real-time quantitative PCR and the 2- ΔΔCT method. Methods. (2001) 25:402–8. doi: 10.1006/meth.2001.1262. PMID: 11846609

[33] Zheng X Feyaerts AF Van Dijck P Bossier P . Inhibitory activity of essential oils against Vibrio campbellii and Vibrio parahaemolyticus. Microorganisms. (2020) 8:1946. doi: 10.3390/microorganisms8121946. PMID: 33302532 PMC7763747

[34] Natrah FM Ruwandeepika HD Pawar S Karunasagar I Sorgeloos P Bossier P . Regulation of virulence factors by quorum sensing in Vibrio harveyi. Veterinary Microbiol. (2011) 154:124–9. doi: 10.1016/j.vetmic.2011.06.024. PMID: 21775075

[35] Food and Agriculture Organization of the United Nations (FAO) . The FAO action plan on antimicrobial resistance 2021–2025: supporting innovation and resilience in food and agriculture sectors [Internet]. Rome: FAO (2021). doi: 10.4060/cb5545en

[36] Defoirdt T . Virulence mechanisms of bacterial aquaculture pathogens and antivirulence therapy for aquaculture. Rev Aquac. (2014) 6:100–14. doi: 10.1111/raq.12030. PMID: 41834780

[37] Lewis K . Persister cells. Annu Rev Microbiol. (2010) 64:357–72. doi: 10.1146/annurev.micro.112408.134306. PMID: 20528688

[38] Vestby LK Grønseth T Simm R Nesse LL . Bacterial biofilm and its role in the pathogenesis of disease. Antibiotics. (2020) 9:59. doi: 10.3390/antibiotics9020059. PMID: 32028684 PMC7167820

[39] Hall-Stoodley L Costerton JW Stoodley P . Bacterial biofilms: from the natural environment to infectious diseases. Nat Rev Microbiol. (2004) 2:95–108. doi: 10.1038/nrmicro821. PMID: 15040259

[40] Nithya C Pandian SK . The *in vitro* antibiofilm activity of selected marine bacterial culture supernatants against Vibrio spp. Arch Microbiol. (2010) 192:843–54. doi: 10.1007/s00203-010-0612-6. PMID: 20697692

[41] Worthington RJ Richards JJ Melander C . Small molecule control of bacterial biofilms. Organic Biomolecular Chem. (2012) 10:7457–74. doi: 10.1039/C2OB25835H. PMID: 22733439 PMC3431441

[42] Van Kessel JC Rutherford ST Shao Y Utria AF Bassler BL . Individual and combined roles of the master regulators AphA and LuxR in control of the Vibrio harveyi quorum-sensing regulon. J Bacteriol. (2013) 195:436–43. doi: 10.1128/jb.01998-12. PMID: 23204455 PMC3554009

[43] Burt SA Ojo-Fakunle VT Woertman J Veldhuizen EJ . The natural antimicrobial carvacrol inhibits quorum sensing in Chromobacterium violaceum and reduces bacterial biofilm formation at sub-lethal concentrations. PloS One. (2014) 9:e93414. doi: 10.1371/journal.pone.0093414. PMID: 24691035 PMC3972150

[44] Tapia-Rodriguez MR Hernandez-Mendoza A Gonzalez-Aguilar GA Martinez-Tellez MA Martins CM Ayala-Zavala JF . Carvacrol as potential quorum sensing inhibitor of Pseudomonas aeruginosa and biofilm production on stainless steel surfaces. Food Control. (2017) 75:255–61. doi: 10.1016/j.foodcont.2016.12.014. PMID: 41847267

[45] Wang J Qin T Chen K Pan L Xie J Xi B . Antimicrobial and antivirulence activities of carvacrol against pathogenic Aeromonas hydrophila. Microorganisms. (2022) 10:2170. doi: 10.3390/microorganisms10112170. PMID: 36363761 PMC9699308

[46] Kim YM Shin M Kang JW Kang DH . Effect of sub‐lethal treatment of carvacrol and thymol on virulence potential and resistance to several bactericidal treatments of Staphylococcus aureus. J Food Saf. (2022) 42:e13004. doi: 10.1111/jfs.13004. PMID: 41834780

[47] Baruah K Ranjan J Sorgeloos P Bossier P . Efficacy of heterologous and homologous heat shock protein 70s as protective agents to Artemia franciscana challenged with Vibrio campbellii. Fish Shellfish Immunol. (2010) 29:733–9. doi: 10.1016/j.fsi.2010.07.011. PMID: 20643210

[48] Xiao B Hou D Pan J Kang F Wang Y He J . Heat shock protein 70 (HSP70) regulates innate immunity and intestinal microbial homeostasis against Vibrio parahaemolyticus in shrimp. Aquaculture. (2025) 596:741814. doi: 10.1016/j.aquaculture.2024.741814. PMID: 41847267

[49] Iryani MT Lv A Sun J Anirudhan A Tan MP Danish-Daniel M . Effects of heat shock protein 70 knockdown on the tolerance of the brine shrimp Artemia franciscana to aquaculture-related stressors: Implications for aquatic animal health and production. Aquaculture. (2022) 550:737872. doi: 10.1016/j.aquaculture.2021.737872. PMID: 41847267

[50] Sung YY Liew HJ Ambok Bolong AM Abdul Wahid ME MacRae TH . The induction of Hsp70 synthesis by non‐lethal heat shock confers thermotolerance and resistance to lethal ammonia stress in the common carp, Cyprinus carpio (Linn). Aquacult Res. (2014) 45:1706–12. doi: 10.1111/are.12116. PMID: 41834780

[51] Baruah K Huy TT Norouzitallab P Niu Y Gupta SK De Schryver P . Probing the protective mechanism of poly-ß-hydroxybutyrate against vibriosis by using gnotobiotic Artemia franciscana and Vibrio campbellii as host-pathogen model. Sci Rep. (2015) 5:9427. doi: 10.1038/srep09427. PMID: 25822312 PMC4378509

[52] Cerenius L Söderhäll K . The prophenoloxidase‐activating system in invertebrates. Immunol Rev. (2004) 198:116–26. doi: 10.1111/j.0105-2896.2004.00116.x. PMID: 15199959

[53] Moreira AC Mesquita G Gomes MS . Ferritin: an inflammatory player keeping iron at the core of pathogen-host interactions. Microorganisms. (2020) 8:589. doi: 10.3390/microorganisms8040589. PMID: 32325688 PMC7232436

[54] Shibata T Kawabata SI . Transglutaminase in invertebrates. In: Transglutaminases: Multiple Functional Modifiers and Targets for New Drug Discovery. Springer Japan, Tokyo (2016). p. 117–27. doi: 10.1007/978-4-431-55825-5_5, PMID:

[55] Anirudhan A Okomoda VT Iryani MT Andriani Y Abd Wahid ME Tan MP . Pandanus tectorius fruit extract promotes Hsp70 accumulation, immune-related genes expression and Vibrio parahaemolyticus tolerance in the white-leg shrimp Penaeus vannamei. Fish Shellfish Immunol. (2021) 109:97–105. doi: 10.1016/j.fsi.2020.12.011. PMID: 33352338

